# Anthropometry-driven block setting improves starting block performance in sprinters

**DOI:** 10.1371/journal.pone.0213979

**Published:** 2019-03-27

**Authors:** Valentina Cavedon, Marco Sandri, Mariola Pirlo, Nicola Petrone, Carlo Zancanaro, Chiara Milanese

**Affiliations:** 1 Department of Neurosciences, Biomedicine and Movement Sciences, University of Verona, Verona, Italy; 2 OMR Automotive, Brescia, Italy; 3 Department of Industrial Engineering, University of Padua, Padua, Italy; University of Colorado Boulder, UNITED STATES

## Abstract

This study tested the effect of two block setting conditions i.e., the usual block setting [US] and an anthropometry-driven block setting [AS] on the kinematic and kinetic parameters of the sprint start. Furthermore, we verified whether this effect is influenced by the relative lengths of the sprinter’s trunk and lower limbs i.e., the Cormic Index by subdividing sprinters into brachycormic, metricormic and macrocormic groups. Forty-two sprinters performed 6 maximal-effort 10 m sprints using the US and AS conditions. Dynamometric starting blocks measured forces generated by the sprinters. The times at 5 m and 10 m in the sprint trials were measured with photocells. Results showed that the anteroposterior block distances were significantly different between the two conditions (P<0.001). Across the sample, the horizontal block velocity, the rear peak force, the rear force impulse, the total force impulse, the horizontal block power, the ratio of horizontal to resultant impulse in the rear block, the first and second step lengths and the times at 5 m and 10 m improved in AS vs. US (P values from 0.05 to 0.001). Considering the interaction between the block setting condition and the Cormic Index, the rear peak force and the rear force impulse were significantly increased in the metricormic and brachycormic groups (P≤0.001) and the metricormic group (P<0.001), respectively. Kinetic variables in the rear block and the difference (Delta) in the front block/starting line distance between US and AS were related with each other (Adjusted R^2^ values from 0.07 to 0.36). In conclusion, AS was associated with improvement in the kinematic and kinetic parameters of the sprint start performance vs. US; however, AS is apparently best suited for metricormic sprinters. Further work is needed to verify how the sprint start kinetic and kinematic parameters are related to the front block/starting line distance and whether a block setting driven by the sprinter’s Cormic Index is able to improve sprint start performance.

## Introduction

In track sprints, the success of the sprint start performance depends on the ability of the sprinter to generate a large impulse over the shortest time and reach the highest running speed as soon as possible [1–3]. This phase is especially important in the 100 m sprint [4–7].

Acceleration at the start of a race is influenced by the way sprinters positions themselves in the blocks at the set command and the mechanics of leaving the blocks at the sound of the gun [8,9]. The kinematic and kinetic patterns of elite athletes during the starting block phase and acceleration phase have received considerable attention and many variables have been studied to account for the effects of starting technique [2,3,5, 9–18].

According to the literature an effective sprint start mainly depends on the start block positioning and the joint angles of the lower limbs in the set position [8,9,11]. Furthermore, the pushing time on the blocks and the forces generated by the front and rear legs in the pushing phase [3,9–18] are also important. Recent studies found that the most predictive factor of sprint start performance was the magnitude of force generated by the rear leg [16,19]. What is more, the average horizontal external power (i.e., the ability to translate the centre of mass in the running direction in a short period of time) was identified as an excellent descriptor of start performance in sprinters [2].

The starting technique is greatly influenced by the setting of the block positions with regards to spacing and obliquities [8,20,21]. Bezodis, Salo, & Trewartha [3] demonstrated that “a single optimal set position” for everybody is not recommended due to varying physical factors, and therefore sprinters generally find their own preferred distance between the blocks according to sensations or outcomes. For example, one of the most popular adjustments frequently modified by the sprinters is the anteroposterior inter-block spacing and finding the optimum setting for each athlete may take a long period of training. Furthermore, athletes may not actually be selecting their ideal block setting for best performance when only basing their preference on sensation.

The importance of anteroposterior inter-block spacing on the sprint movement during the block start phase has been extensively investigated in several studies [3,6,9,12, 21–24]. The three main types of block spacing investigated in the literature [20] are the bunched start (spacing generally <30 cm), the medium start (30 to 50 cm) and the elongated start (>50 cm). A number of studies found that the velocity of the centre of mass at block clearing is higher when the inter-block spacing increases due to an increase of force impulse [2,11,22]. This is due to an increased duration of force generated against the blocks and a greater contribution of total force impulse from the rear leg [21,25–27]. What is more, an increase in force generation from the rear leg has been associated with higher block clearing velocities in elite sprinters [7,14,18]. However, a recent study [12] demonstrated that in the elongated start, despite a greater velocity of the center of mass at block clearing, the performance at 5 m and 10 m is significantly worse compared to the bunched start. It is known that in the elongated start, the duration of force application is increased during the block phase [9]. Spending longer on the blocks increases the total run time which then conflicts with the objective of a sprint. Instead, at 10 m the best performance results were obtained from the medium start. Further, a number of studies [3,9,12,22–24] suggested that the medium start creates the best balance between total force generated and the increased time of force generation to obtain the best performance in the early acceleration phase.

When looking for the best front block/starting line and inter-block distances for an individual athlete, it would seem obvious that the block distances should be relative to the individual’s body dimensions. However, very few studies considered the anteroposterior block distances in relation to the individual body dimensions of the athlete [11,20,22]. A study conducted by Dickinson [20] stated that the distance of the front block from the starting line should depend on the height of the individual, and the distance of the rear block from the starting line on the leg and the thigh lengths, irrespective of the type of the start used (bunched, medium, elongated). Henry [22] investigated if the optimum block spacing is related to the individual leg length by analyzing the interaction between four experimental inter-block distances (27.9, 40.6, 53.3 and 66.0 cm) and the individual’s leg length. Contrary to Dickinson, Henry concluded that leg length is not important in determining the best block spacing. A later study conducted by Schot & Knutzen [11] went further in associating the athlete’s physical characteristics with the anteroposterior block spacing. In this study, the authors stated that a medium start could be achieved according to a calculation based on the individual’s leg length from the greater trochanter to the lateral malleolus (60% of this length was used as the front block/starting line distance and 45% for the inter-block spacing).

Anthropometry has been shown to play an important role in sports where body proportionality differences may affect the biomechanics of movement and the resulting performance (e.g. in running and in gymnastics) [28,29]. However, there is a lack of research addressing anthropometric characteristics in the sprint start. Accordingly, research is needed to elucidate any connection between the physical characteristics of the athlete, the block settings and the kinematic and kinetic parameters during the sprint start. In starting block performance, it is reasonable to assume that body proportionality would influence optimal anteroposterior block distances. The starting block performance involves a closed kinematic chain of movements where the body extremities are in a fixed position and the only modifiable parameters are the anteroposterior block distances. Thus, it can be argued that, in addition to body dimensions, the proportion between the leg and trunk length may also affect the optimal anteroposterior block distances. It is well known that individuals present different proportionality characteristics between the leg and the trunk lengths and a way to assess such a proportionality is the Cormic Index [30]. The Cormic Index expresses sitting height as a proportion of the total height, representing a measure of the relative lengths of the trunk and lower limbs. Individuals are classified as brachycormic, metricormic and macrocormic according to a Cormic Index ≤51%, 51–53%, or ≥53%, respectively [31].

Using instrumented starting blocks and high speed video cameras, the first aim of this study was to test the effect of two different block settings in terms of anteroposterior block distances on the kinematic and kinetic parameters of well-trained sprinters. The two setting conditions were the usual personal block setting used by the athlete and a block setting based on a proportion of the individual’s leg length [11]. We hypothesized that an anthropometry-driven intervention may improve the sprint start performance outcome. The second aim was to verify whether an effect of the two block settings persists when the Cormic Index of the participants is considered. The body proportionality characteristics of the sprinters may be of relevance in a sprint start, a skill where the entire body is involved in a closed chain of movements. We hypothesized that the Cormic Index of participants could influence the effect of a block setting based on the leg length. It is expected that results of this work would help coaches and athletes to improve sprint start performance using a quick anthropometry-driven procedure.

## Materials and methods

### Participants

The required sample was estimated “a priori” and calculated using G*Power ver.3.1.9.2 [32]. Setting the type I error [SS3] at α = 0.05, the effect size at f = 0.25 and the correlation among repeated measures at 0.6, the minimum sample size required for a within-between interaction in a mixed-design ANOVA for having an 80% power (i.e., β = 0.20) was 36 subjects. In order to comply with a possible ~15% dropout, forty-two participants were initially recruited. Participants were well-trained skilled sprinters (20 women and 22 men) with a competitive athletic career of at least 2 years in sprint running. Female and male participants’ age, height and body mass (±SD) were 19.70 ± 2.23 and 19.36 ± 2.11 y, 165.4 ± 5.2 and 176.7 ± 5.9 cm, 55.6 ± 6.8 and 67.1 ± 9.8 kg, respectively. All sprinters were involved in regional and national level competitions and trained at least 6 times a week for 2/3 hours per day. Their best time over 100 m ranged between 10.45 s and 11.30 s for men and between 11.45 s and 12.68 s for women. According to the Cormic Index, the sprinters were brachycormic (n = 12), metricormic (n = 19) and macrocormic (n = 11). The mean Cormic Index in the three groups was 50.62% ± 1.14, 52.48% ± 0.98 and 53.78% ± 0.51, respectively. All participants gave their written informed consent to participate in the study, and the protocol was performed in accordance with the Declaration of Helsinki. Ethics approval was obtained from the University of Verona Institutional Review Board.

### Experimental procedure

The sprint testing took place on an outdoor track (Olimpic Plast SWD surface, Olimpia Costruzioni, Forlì, Italy) during the early competition phase of the outdoor season. One operator (VC) attached ten retro-reflective passive flat markers (14 mm diameter) bilaterally over specific anatomical landmarks on the participant’s body (i.e., right [R] and left [L] acromion, R and L femur greater trochanter, R and L femur lateral epicondyle, R and L fibula apex of lateral malleolus, R and L 5^th^ metatarsal).

Following a warm-up consisting of jogging, dynamic stretching and sprints of submaximal intensity, all participants performed a total of 6 maximal-effort 10 m sprints using two different starting conditions: 1) the athlete’s usual personal block setting (US), and 2) an anthropometry-driven setting (AS). The order of the two starting conditions was randomized for each athlete. In the AS condition, the start block positions were set according to a calculation based on the individual’s leg length from the greater trochanter to lateral malleolus [11]; 60% of this length was used as the front block/starting line distance and 45% as the inter-block spacing. The anteroposterior block distances in both the US and AS conditions were measured to the nearest 0.1 cm in the outdoor track with a fiberglass tape. The obliquity of the blocks was that usually used by the participants and was the same in both conditions. Participants used their own spiked shoes for sprint running. For all trials, each sprint was initiated by the same experimenter (MP), who provided standard ‘on your marks’ and ‘set’ commands. The experimenter then pressed a custom-designed trigger button to provide the auditory start signal through a sounder device. The rest period between trials was 5–7 minutes.

After an adequate rest period, the standing long jump test was used to measure lower extremity strength. Participants stood behind a line marked on the ground with their feet slightly apart. A two-foot take-off and landing was used, with swinging of the arms and bending of the knees to provide forward drive, the subject attempting to jump as far as possible. The horizontal distance between the starting line and the back of the heel closest to the starting line at landing was recorded via tape measure to the nearest 0.1 cm. Three trials were performed (with adequate rest between trials) and the maximum distance was recorded. Although the standing long jump performance is usually expressed in absolute terms as the overall distance covered, it has been suggested that the subject’s leg length can play a significant role in the performance [33]. Accordingly, performance in the standing long jump test was expressed relative to the leg length (SLJ-relative).

### Data collection

#### Anthropometric data

Anthropometric data were taken by one operator (VC) using conventional criteria and measuring procedures [34]. Body mass was assessed to the nearest 0.1 kg using a certified electronic scale (Tanita electronic scale BWB-800 MA, Wunder SA.BI. Srl, Milano, Italy). Standing height and sitting height were measured to the nearest 0.1 cm using a Harpenden portable stadiometer (Holtain Ltd., Crymych, Pembs. UK). For the sitting height, the subject was asked to sit on a flat stool of a known height. Measurement was taken with the subject sitting in a standard position. The sitting height was then obtained by subtracting the height of the stool from the reading on the stadiometer. The lower limb length was measured with a Harpenden anthropometer (Holtain Ltd., Crymych, Pembs. UK) as the distance between the greater trochanter and the lateral malleolus (cm). Body circumferences were measured with a fiberglass tape at the mid-thigh and the calf. The Cormic Index was calculated for each participant as sitting height (cm)/standing height (cm)∙100. The body mass index (BMI) was also computed for each participant as body mass (kg)/standing height (m)^2^.

#### Kinetic and kinematic data

**Fig 1:**
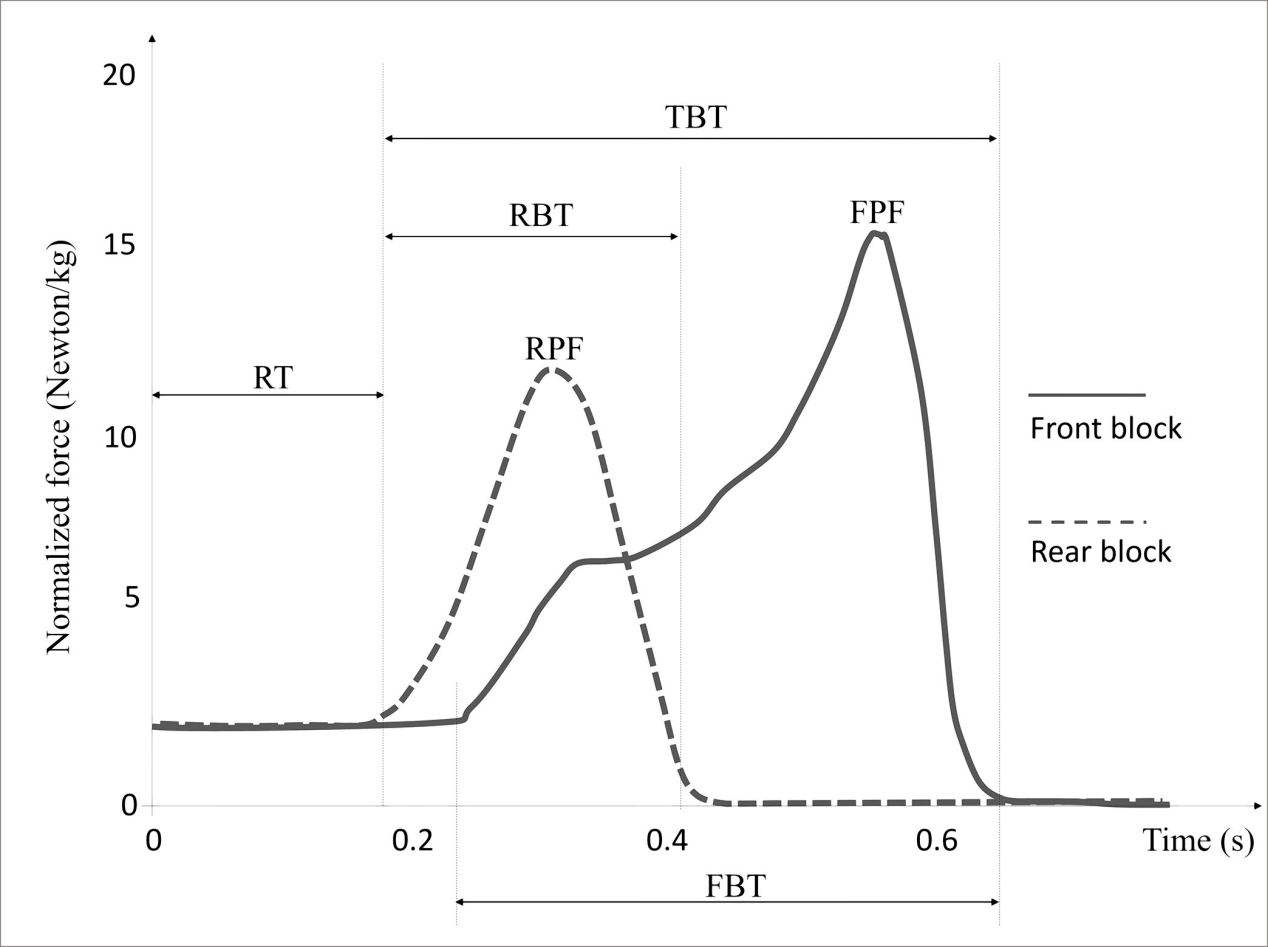
Example of resultant force curves of the front and rear blocks during a sprint start recorded by the instrumented starting blocks. RT, reaction time; RBT, rear block time; FBT, front block time; TBT, total block time; RPF, rear peak force; FPF, front peak force.

Each trial was performed using a set of dynamometric starting blocks equipped with a set of load cells (CU K5D and CU K1C models, GEFRAN SpA, Brescia, Italy) enabling the measurement of the magnitude and direction of forces generated by the sprinter during the starting block phase. The acquisition frequency was 1 kHz and the sensitivity was 0.01 N. These blocks respected all the features of those normally used in the track sprint start, as well the output characteristics of similar blocks used in previous works [13,16]. There were four monoaxial load cells installed on each block, which were used to determine the loads: three cells were used to measure the vertical loads and one to measure the horizontal loads. The force data were resolved into horizontal and vertical components for each foot (Fig 1). The blocks were connected to a portable personal computer (SoftPLC, GEFRAN SpA, Brescia, Italy) that stored data, and handled signal processing and parameter calculation. The dynamometric starting blocks were developed at OMR Automotive (Officine Meccaniche Rezzatesi, Brescia, Italy).

The reliability and validity tests were performed at the Department of Industrial Engineering, University of Padua, Italy. Both static and dynamic bench tests were carried out on a mechanical bench equipped with two MTS 242 servo hydraulic actuators to calibrate the force transducers. Several tests were performed in the horizontal and vertical directions on each part of the apparatus in order to obtain the single axis calibration constants of the system. Static and dynamic load tests with combined loading were also performed to validate the results of a realistic usage, showing good results with low error: the difference between the maximum force applied on the block and the measured force was lower than 1% [35]. After bench tests, a series of in-vivo sprint starts were recorded during a training session with beginner, intermediate and expert athletes and comparison was made with a commercial force plate (Model 4060–10, Bertec Columbus, OH, USA) output [35] included in a SMART DX-6000 motion capture system (BTS Bioengineering, Quincy, MA, USA) after mounting the dynamometric blocks over the force platform. The results showed that both vertical and horizontal forces measured by the dynamometric starting blocks and the force plate were very similar to each other: horizontal and vertical forces were able to follow the loads also in the region of rapid loading and unloading with good accuracy. Regression coefficients were found to be on average R = 0.9946 for horizontal loads and R = 0.9978 for vertical loads. The dynamometric starting blocks apparatus was portable and made resistant to splash water and could therefore be used outdoors. These dynamometric starting blocks can easily be used on the track during training, giving precise quantitative data which would usually only be available in a biomechanics laboratory setting using sophisticated instruments in a controlled environment.

**Fig 2:**
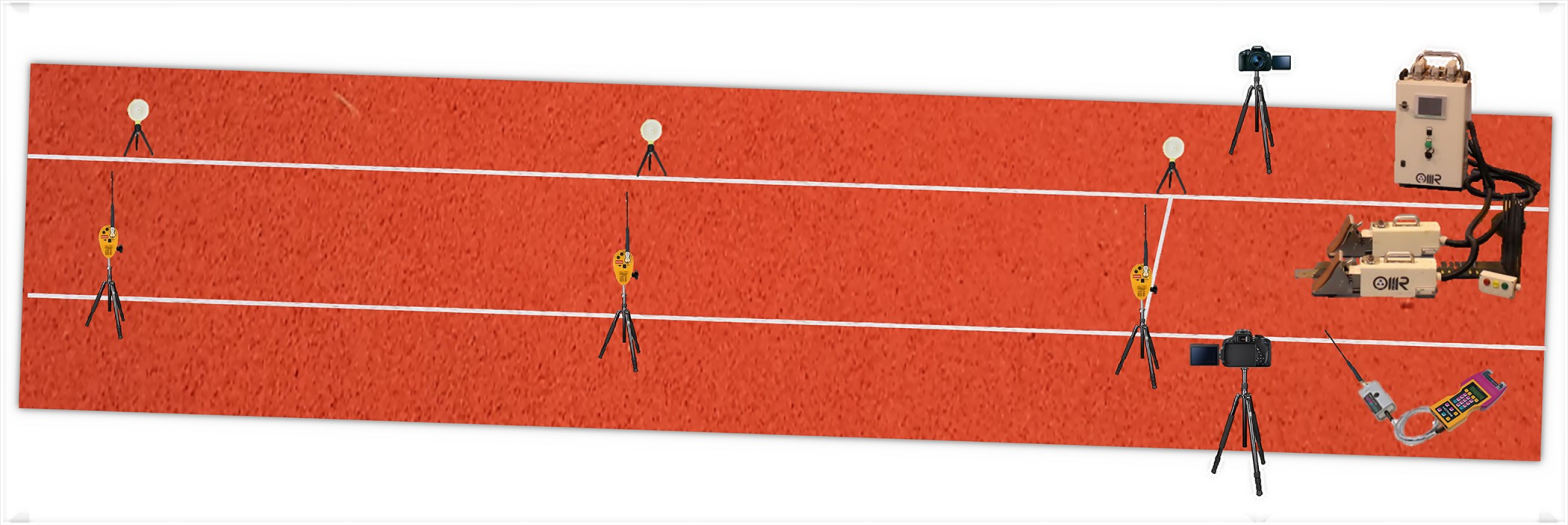
Experimental set-up.

Two high-speed video cameras (Casio Exilim ex-zr 1000, Casio Europe Gmbh, Barcelona, Spain) captured the movement of each athlete in two dimensions during the starting block and acceleration phases (first and second stride lengths). One camera (Camera 1) was positioned for the front block side view of the participant and the other camera (Camera 2) for the rear block side view of the participant. According to Bartlett [36], in order to limit the potential technological errors in the filming set-up connected with the 2D videography (e.g. the parallax errors due to visual distortion), the optical axis of each camera was lined directly perpendicular to the sprinter’s movement plane. Furthermore, the optical axis of each camera was centred in correspondence to an imaginary line perpendicular to the ground and passing through the hip joint of the leg facing the camera at the “on your marks” position. Each camera was placed on a tripod at a height of approximately 1 m and located at approximately 5 m from the participant. Each camera’s field of view provided a sagittal view of each sprinter for the first two full strides. The position of the two cameras was standardized for all participants to ensure no environmental changes during field testing (Fig 2). Each video was calibrated with a 50 cm cube positioned to the rear of the blocks and defined by X the horizontal axis and Y the vertical axis. Images of the starting block phase and acceleration phase were collected at a resolution of 1280 x 1024 pixels using a shutter speed of 1/1000 s and a sampling frequency of 200 Hz.

Three pairs of photocells (Polifemo Light Radio, Microgate SRL, Bolzano, Italy) based on a radio impulse transmission system and a reflection system were used to measure the times at 5 m and 10 m in the sprint trials. The timing between the dynamometric starting blocks and the photocells system was synchronized using the digital output available from the block control system and connecting it to the available input for timing available in the Microgate unit.

#### Data analysis

The kinematic and kinetic outputs were stored during the trials in a portable personal computer connected to the instrumented blocks and were then exported for further analysis. Raw data were filtered using a low-pass Butterworth filter (fourth order) with a cutoff frequency of 120 Hz and analyzed using a custom program written in Matlab R2008a (MathWorks, Natick, MA, USA).

Force signals were resolved into horizontal and vertical components using a coordinate system affixed to the runway. The x-axis pointed forward along the running surface (horizontal plane), the y-axis pointed vertically upwards. Force data were used to define the force onset threshold (i.e., when the first derivative of the resultant force-time curve was greater than 500 Ns^-1^) and the force offset thresholds (i.e., when the resultant force was lower than 50 N). The pushing phase was defined between the first instant of block start (i.e., corresponding to the force onset threshold) to block clearance (i.e., corresponding to the force offset threshold on the front block). The pushing phases of the front and the rear blocks were defined as the period between the instant of the block start and the end of the respective sub-phases for each block. The following temporal parameters were extracted for analysis from the instrumented blocks data: the reaction time (RT), defined as the time from the auditory signal to the first force onset threshold; the front block time (FBT), defined as the pushing time on the front block sub-phase; the rear block time (RBT), defined as the pushing time on the rear block sub-phase and the total time block (TBT), defined as the pushing time on the total pushing phase. The following kinetic variables obtained from the instrumented blocks data during the pushing phase were also measured: the front peak force (FPF), defined as the maximum resultant front force value; the rear peak force (RPF), defined as the maximum resultant rear force value; the horizontal and the vertical front peak force (H_FPF and V_FPF); the horizontal and the vertical rear peak force (H_RPF and V_RPF); the average total force (ATF). The front force impulse (FF_impulse_), the rear force impulse (RF_impulse_) and the total force impulse (Total F_impulse_) were computed according to Otsuka and colleagues [15]. All the kinetic variables were normalized to the body mass of the sprinters expressed in kg. In addition, the following variables were computed: the ratio of horizontal to resultant force impulse of both legs (Ratio_front and Ratio_rear) [37]; the horizontal block velocity (H_BV) measured as the sum between the horizontal impulse on the front block plus the horizontal impulse on the rear block (both expressed in Ns) divided by the body mass of the sprinter expressed in kg; the normalized average horizontal external block power (NAHEP) calculated according to the procedures by Bezodis and colleagues [2].

**Fig 3:**
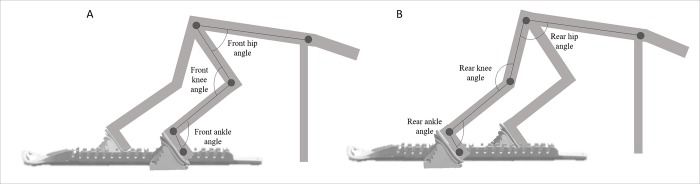
Two-dimensional functional representation of the joint angles on the front (Panel A) and rear blocks (Panel B).

For each participant the video material of the 12 video clips (6 trials and 2 cameras) was uploaded onto a PC and digitalized at full resolution with a zoom factor of 2.5 using freeware motion-analysis software (Kinovea; version 0.8.15, available for download at: http://www.kinovea.org). One operator (VC) blinded to condition (AS, US) manually digitalized the markers and quantified the joint angles and stride lengths at specific video frames on each video (see details below). The analysis of the video material was carried out in two stages. Firstly, each video clip was rewound frame by frame and frozen at the set position to directly estimate the angles in the sagittal plane with the digital goniometer built into the Kinovea software. The operator visually digitalized the markers through the “cross marker” function and placed the digital goniometers. The videos from Camera 1 (i.e., with the front block side view) were used to estimate the hip, knee and ankle joint angles on the front leg while the videos from Camera 2 (i.e., with the rear block side view) were used to measure the hip, knee and ankle joint angles on the rear leg (Fig 3). The joint angles were measured to the nearest 1 degree. Secondly, in order to measure the length of the first stride, each video clip from Camera 1 was stopped at the instant of the first foot strike. Stride was estimated at the first frame of the video clip where the sprinter’s foot made contact with the track surface. The length of the inferior side of the cube positioned to the rear of the blocks in the frame was used as a reference to calibrate all other line lengths. The “line drawing tool” function was used to assess the horizontal distance between the front block and the toe of the rear foot at the first foot strike (first stride length [SL_1_]). Thirdly, in order to measure the length of the second stride, each video clip from Camera 2 was stopped at the instant of the second foot strike. The “line drawing tool” function was used to assess the horizontal distance to the nearest 0.1 cm between the rear foot at the first toe off and toe of the front foot at the first foot strike (second stride length [SL_2_]). SL_1_ and SL_2_ were normalized to leg length to account for differences among subjects and labelled as NorSL_1_ and NorSL_2,_ respectively.

In order to limit any technical errors involved in 2D videography as much as possible, the procedure adopted to quantify the joint angles and stride lengths was repeated in three separate sessions, with a minimum interval of 7 days between sessions and the mean value was recorded only when the coefficient of variation was <0.05. Furthermore, the operator was familiar with the use of high-speed video as a tool to quantify joint angles in sprint running and in sprint starts. It has been noted that the markers, despite being properly positioned during data collection, can move in relation to the skin throughout the range of motion [38]. Accordingly, in line with Bradshaw and colleagues [39], the operator paid close attention during data analysis to this fact and visually adjusted for skin movement by only using the markers as a guide.

In this study, errors (i.e. parallax and lens distortion errors) in the measurement of the first and second stride lengths has to be taken into account. A typical error (%Error) was estimated starting from the calibration cube (50 cm) positioned behind the blocks and then every 50 cm until the end of the field of view. The %Error was calculated according to the following formula:

%Error=|E−A|/A·100

where *A* was the real value corresponding to the 50 cm distance measured on the field with a measuring tape; *E* was the experimental measure corresponding to the threefold measurement of the same distance as above in Kinovea with the “line drawing tool” function. In the experimental setup used in this study, errors in the measurement of the first and second stride lengths are expected due to parallax and lens distortion errors. Actually, the percent error in the measurement of a distance of 50 cm at 1.0 and 3.0 meters from the calibration cube was about 1.3% and 3.7%, respectively. However, the error was reproducible for any measurement, thereby not affecting the comparison of the two investigated starting block conditions.

#### Statistical analysis

In this work an AB/BA crossover design was adopted. Data were assessed for normality with the Kolmogorov-Smirnov test and transformed using the method described by Box and Cox [40] where necessary. The Box and Cox transformation provides an algorithm through which the optimal value of the transformation parameter λ is selected by the method of maximum likelihood for reducing heterogeneity of error that permits the assumption of equal variance to be met. The equal variance assumption was checked using the Levene’s tests. Descriptive statistics (mean and standard deviation) were computed for all variables using standard procedures. Comparison of variables in the three Cormic Index groups (brachycormic, metricormic, and macrocormic) was carried out with one-way ANOVA followed where needed by the post hoc test with Bonferroni’s correction for multiple comparisons. A mixed-design ANOVA with the three groups (brachycormic, metricormic, macrocormic) and the two block setting conditions (US and AS) with repeated measures on the second factor was performed to assess the effect of different block settings on kinetic and kinematic variables and its interaction with the sprinters’ Cormic Index. When the repeated measures factor violated the assumption of sphericity (P<0.05), the Greenhouse-Geisser correction, which refers to degrees of freedom of F statistics, was used. For each ANOVA model, if significant interactions were detected (group by condition) post hoc analysis with Bonferroni’s correction was carried out. Cohen’s partial eta squared (Ƞ_p_^2^) was used to calculate the effect size in the ANOVA with repeated measures. According to Cohen’s guidelines [41], effect size values were interpreted as small (Ƞ_p_^2^ = 0.01), medium (Ƞ_p_^2^ = 0.06), and large (Ƞ_p_^2^ = 0.14).

For each performance variable, a multiple linear regression model was estimated using the difference (Delta) in the anteroposterior block distances between the two conditions (US and AS) as predictors and the Delta in the performance variable as the dependent variable. The adjusted coefficient of determination (Adj. R^2^) was used as a goodness-of-fit measure for the model. The strength of the relationship between the outcome and a single predictor, after excluding the effect of the other predictors, was estimated by partial correlation. Statistical analysis was carried out using SPSS v.16.0 (IBM Corp., Armonk, NY) and the statistical significance was set at p ≤ 0.05.

## Results

The characteristics of the participants in the whole sample as well as the three Cormic Index groups are summarized in Table 1. The three groups were similar (one-way ANOVA; P>0.05) for age, several anthropometric measurements, sprint start experience, and standing long jump performance expressed relative to the leg length. As expected, significant differences were found for the sitting height and the lower limb length (F = 13.238 and F = 14.495, respectively; P<0.001 for both). Post-hoc analysis with Bonferroni’s correction revealed that the brachycormic group had significantly lower sitting height and lower limb length values in comparison with both the metricormic (P = 0.002 for both) and the macrocormic (P<0.001 for both) groups (Table 1). The mean values (±SD) of the anteroposterior block distances and the kinematic and kinetic data in the US and AS conditions in the whole sample and in the three Cormic Index groups are reported in Table 2.

**Table 1:** Characteristics of the participants in the whole sample and the three Cormic Index groups. Data are means ± SD.

	Whole sample (n = 42)	Brachycormic (n = 12)	Metricormic (n = 19)	Macrocormic (n = 11)
Age (y)	19.52 ± 2.14	19.75 ± 2.38	19.63 ± 2.41	19.09 ± 1.38
Sprinting experience (y)	4.76 ± 2.67	5.83 ± 3.04	3.95 ± 2.27	5.00 ± 2.65
Body mass (kg)	61.6 ± 10.2	59.6 ± 9.9	63.7 ± 10.4	60.3 ± 10.4
Height (cm)	171.3 ±7.9	167.6 ± 8.1	172. 4 ± 6.8	173.6 ± 8.8
BMI (kg/m^2^)	20.89 ± 2.19	21.10 ± 1.98	21.33 ± 2.44	19.89 ± 1.76
Sitting height (cm)	89.6 ± 5	84.9 ± 5*	90.4 ± 3	93.33 ± 4.6
Lower limb length (cm)	81.8 ± 5	77.1 ± 5*	82.5 ± 2.8	85.8 ± 4.2
Thigh circumference (cm)	49.04 ± 3.27	49.57 ± 3.97	49.32 ± 3.38	47.98 ± 2.09
Calf circumference (cm)	34.72 ± 2.56	34.73 ± 3.08	35.01 ± 2.41	34.22 ± 2.35
SLJ-relative	2.8 ± 0.2	2.7 ± 0.2	2.9 ± 0.3	2.7 ± 0.2

BMI, body mass index; SLJ-relative, performance in the standing long jump test expressed relative to the leg length.

*, significantly different (P<0.05; Bonferroni’s post-hoc) vs. metricormic and macrocormic.

**Table 2:** Block distances, and kinematic and kinetic data in the sprint start for the whole sample as well as for the three Cormic Index groups in their usual block setting condition (US) and an anthropometry-driven block setting (AS) condition. Data are means ± SD.

Variable	Whole sample (n = 42)		Brachycormic (n = 12)		Metricormic (n = 19)		Macrocormic (n = 11)	
**Block distances**	US	AS	US	AS	US	AS	US	AS
FB/SL distance (cm)	52.3 ± 4.8	49.1 ± 3.0*	48.0 ± 3.6	46.3 ± 3.1	53. 9 ± 4.3	49.5 ± 1.7	54.2 ± 3.9	51.5 ± 2.5
I-B distance (cm)^	27.6 ± 2.4	36.8 ± 2.3*	27.3 ± 2.5	34.7 ± 2.3^#^	27.2 ± 2.2	37.1 ± 1.3^#^	28.6 ± 2.5	38.6 ± 1.9^#^
**Set position**								
Front hip (°)	47 ± 6	43 ± 6*	44 ± 6	41 ± 7	49 ± 4	43 ± 4	47 ± 9	44 ± 8
Rear hip (°)	77 ± 8	84 ± 8*	75 ± 8	80 ± 10	79 ± 7	85 ± 5	77 ± 11	85 ± 10
Front knee (°)	92 ± 9	90 ± 8*	94 ± 7	93 ± 5	92 ± 9	88 ± 8	92 ± 9	90 ± 9
Rear knee (°)	112 ± 11	117 ± 11*	115 ± 7	119 ±8	111 ± 12	115±11	112 ± 12	118 ± 15
Front ankle (°)	92 ± 6	93 ± 7	94 ± 4	95 ± 3	92 ± 7	93 ± 7	92 ± 6	92 ± 8
Rear ankle (°)	87 ± 6	85 ± 7*	88 ± 5	86 ± 6	88 ± 6	86 ± 8	85 ± 6	84 ± 7
**Pushing phase**								
RT (s)	0.185 ± 0.035	0.189 ± 0.035	0.195 ± 0.026	0.192 ± 0.029	0.184 ± 0.040	0.189 ± 0.032	0.175 ± 0.035	0.186 ± 0.047
FBT (s)	0.402 ± 0.041	0.416 ± 0.064*	0.402 ± 0.051	0.402 ± 0.044	0.394 ± 0.036	0.419 ± 0.086	0.419 ± 0.036	0.424 ± 0.027
RBT (s)	0.211 ± 0.041	0.212 ± 0.041	0.220 ± 0.052	0.225 ± 0.059	0.203 ± 0.038	0.210 ± 0.035	0.215 ± 0.031	0.202 ± 0.025
TBT (s)	0.421 ± 0.047	0.427 ± 0.038	0.411 ± 0.035	0.415 ± 0.028	0.409 ± 0.035	0.424 ± 0.036	0.451 ± 0.066	0.448 ± 0.045
FPF (N/kg)	16.50 ± 2.27	16.60 ± 2.09	15.18 ± 2.16	15.31 ± 1.80	17.13 ± 2.20	17.16 ± 2.29	16.85 ± 2.04	17.03 ± 1.44
RPF (N/kg)^	11.44 ± 2.48	13.07 ± 3.37*	10.83 ± 3.10	12.74 ± 3.10^#^	11.76 ± 2.03	14.08 ± 2.76^#^	11.55 ± 2.58	11.68 ± 4.24
H_FPF (N/kg)	6.02 ± 0.71	5.91 ± 0.65	5.65 ± 0.74	5.62 ± 0.67	6.25 ± 0.70	6.02 ± 0.71	6.02 ± 0.56	6.06 ± 0.45
V_FPF (N/kg)^	6.13 ± 0.92	6.12 ± 0.90	6.01 ± 0.98	5.91 ± 1.20	6.25 ± 0.87	5.99 ± 0.74	6.06 ± 0.98	6.58 ± 0.68^#^
H_RPF (N/kg)^	4.52 ± 1.09	4.95 ± 1.34*	4.13 ± 1.17	4.58 ± 1.21^#^	4.70 ± 0.97	5.41 ± 1.00^#^	4.63 ± 1.20	4.55 ± 1.78
V_RPF (N/kg)^	3.78 ± 1.12	3.96 ± 1.20	3.36 ± 1.04	3.55 ± 1.07	3.76 ± 1.08	4.23 ± 1.00^#^	4.28 ± 1.16	3.94 ± 1.58
ATF (N/kg)	11.37 ± 1.19	11.55 ± 1.12	11.12 ± 1.58	11.22 ± 1.51	11.63 ± 0.91	11.83 ± 0.87	11.21 ± 1.14	11.44 ± 0.96
FF_impulse_ (Ns/kg)	3.48 ± 0.50	3.56 ± 0.55	3.32 ± 0.61	3.32 ± 0.62	3.52 ± 0.48	3.57 ± 0.58	3.60 ± 0.38	3.79 ± 0.33
RF_impulse_ (Ns/kg)^	1.29 ± 0.40	1.39 ± 0.42*	1.25 ± 0.47	1.34 ± 0.40	1.25 ± 0.30	1.48 ± 0.34^#^	1.40 ± 0.47	1.31 ± 0.56
Total F_impulse_ (Ns/kg)	4.76 ± 0.55	4.93 ± 0.56*	4.56 ± 0.69	4.64 ± 0.65	4.75 ± 0.45	5.01 ± 0.54	5.01 ± 0.51	5.10 ± 0.38
Ratio_front	0.69 ± 0.05	0.69 ± 0.05	0.68 ± 0.05	0.69 ± 0.06	0.70 ± 0.05	0.70 ± 0.05	0.70 ± 0.05	0.67 ± 0.04
Ratio_rear	0.75 ± 0.05	0.76 ± 0.05*	0.75 ± 0.06	0.76 ± 0.06	0.76 ± 0.05	0.77 ± 0.04	0.71 ± 0.03	0.73 ± 0.05
Ratio_total	0.71 ± 0.04	0.72 ± 0.04	0.71 ± 0.05	0.71 ± 0.05	0.72 ± 0.05	0.73 ± 0.04	0.70 ± 0.03	0.69 ± 0.04
H_BV (m/s)	3.36 ± 0.35	3.50 ± 0.39*	3.18 ± 0.41	3.27 ± 0.36	3.40 ± 0.28	3.62 ± 0.38	3.48 ± 0.35	3.52 ± 0.34
NAHEP	0.47 ± 0.90	0.50 ± 0.10*	0.44 ± 0.10	0.46 ± 0.09	0.49 ± 0.08	0.54 ± 0.10	0.46 ± 0.08	0.47 ± 0.08
**Acceleration Phase**								
5 m (s)	1.34 ± 0.10	1.29 ± 0.09*	1.36 ± 0.10	1.31 ± 0.12	1.33 ± 0.10	1.28 ± 0.09	1.33 ± 0.09	1.29 ± 0.06
10 m (s)	2.07 ± 0.13	2.03 ± 0.13*	2.12 ± 0.14	2.09 ± 0.16	2.05 ± 0.15	2.00 ± 0.12	2.06 ± 0.10	2.01 ± 0.07
NorSL_1_	1.09 ± 0.11	1.12 ± 0.12*	1.06 ± 0.09	1.12 ± 0.12	1.11 ± 0.14	1.14 ± 0.15	1.07 ± 0.09	1.07 ± 0.07
NorSL_2_	1.15 ± 0.14	1.19 ± 0.12*	1.12 ± 0.15	1.16 ± 0.11	1.18 ± 0.15	1.12 ± 0.13	1.14 ± 0.10	1.15 ± 0.10

FB/SL, front block/starting line; I-B, inter-block; RT, reaction time; FBT, front block time; RBT, rear block time; TBT, total block time; FPF, front peak force; RPF, rear peak force; H_FPF, horizontal front peak force; V_FPF, vertical front peak force; H_RPF, horizontal rear peak force; V_RPF, vertical rear peak force; ATF, average total force; FF_impulse_, front force impulse; RF_impulse_, rear force impulse; Total F_impulse_, total force impulse; Ratio_front, Ratio of horizontal to resultant force impulse of front leg; Ratio_rear, Ratio of horizontal to resultant force impulse of rear leg; NAHEP, normalized average horizontal external power; H_BV, horizontal block velocity; 5 m, time at 5 meters; 10 m, time at 10 meters; NorSL_1_, first stride length normalized to leg length; NorSL_2_, second stride length normalized to leg length.

*, statistically significant (P<0.05) difference between the AS and US conditions

^, statistically significant (P<0.05) difference of the AS vs. the US effect across the three Cormic Index groups

^#^, statistically significant difference between the AS and US conditions (post-hoc analysis with Bonferroni’s correction).

One-way ANOVA showed that in the US condition, the front block/starting line distance was significantly different within the three groups (F = 9.500, P<0.001), but the inter-block distance was not. Post-hoc analysis with Bonferroni’s correction revealed a significantly lower front block/starting line distance in the brachycormic group vs. the metricormic group (-5.9 cm, P = 0.001) and macrocormic group (-6.1 cm, P = 0.002). One-way ANOVA also indicated that all of the measured sprint start kinematic and kinetic variables were similar in the US condition in the three Cormic Index groups with the exclusion of the TBT (F = 3.369, P = 0.045). However, post-hoc analysis with Bonferroni’s correction, revealed no significant group-group difference in the TBT.

A mixed-design ANOVA with three groups (brachycormic, metricormic, macrocormic) and two block setting conditions (US and AS) with repeated measures on the second factor showed a significant main effect of condition for both anteroposterior block distances and for several kinematic and kinetic measurements (Tables 2 and 3).

**Table 3:** Results of the mixed-design ANOVA to quantify the effect of condition and effect of group by condition interaction on block distances, and kinematic and kinetic data.

	Condition			Group by condition		
Variable	F value	P value	η_p_^2^	F value	P value	η_p_^2^
**Block distances**						
FB/SL distance	29.232	<0.001	0.428	2.308	0.113	0.106
I-B distance	545.112	<0.001	0.933	4.494	0.018	0.187
**Set position**						
Front hip	46.494	<0.001	0.544	2.392	0.105	0.109
Rear hip	60.857	<0.001	0.609	1.520	0.231	0.072
Front knee	11.569	0.002	0.229	1.224	0.305	0.059
Rear knee	10.482	0.002	0.212	1.643	0.531	0.032
Front ankle	3.830	0.058	0.089	0.213	0.809	0.011
Rear ankle	7.485	0.009	0.161	0.150	0.861	0.008
**Pushing phase**						
RT	0.925	0.342	0.023	0.621	0.543	0.031
FBT	4.261	0.046	0.098	1.201	0.312	0.058
RBT	0.031	0.862	0.001	2.661	0.083	0.120
TBT	1.647	0.207	0.041	1.770	0.184	0.083
FPF	0.356	0.554	0.009	0.066	0.937	0.003
RPF	25.821	<0.001	0.398	5.402	0.008	0.217
H_FPF	1.969	0.168	0.048	2.247	0.119	0.103
V_FPF	0.274	0.603	0.007	4.935	0.012	0.202
H_RPF	10.706	0.002	0.215	4.436	0.018	0.185
V_RPF	1.299	0.261	0.032	6.770	0.003	0.258
ATF	2.237	0.143	0.054	0.165	0.848	0.008
FF_impulse_	1.950	0.171	0.048	0.815	0.450	0.040
RF_impulse_	6.821	0.013	0.149	10.191	<0.001	0.343
Total F_impulse_	11.838	0.001	0.233	2.255	0.118	0.104
Ratio_front	1.321	0.257	0.033	3.241	0.050	0.143
Ratio_rear	9.244	0.004	0.192	0.829	0.444	0.041
Ratio_total	0.008	0.930	0.000	1.803	0.178	0.085
H_BV	11.946	0.001	0.234	3.107	0.056	0.137
NAHEP	6.815	0.013	0.149	1.446	0.248	0.069
**Acceleration phase**						
5 m	27.802	<0.001	0.416	0.273	0.762	0.014
10 m	19.531	<0.001	0.334	0.219	0.804	0.011
NorSL_1_	12.041	0.001	0.236	3.002	0.061	0.133
NorSL_2_	12.732	0.001	0.246	1.241	0.300	0.060

US, usual sprinter’s block setting; AS, anthropometry-driven block setting; η_p_^2^, partial eta squared; FB/SL, front block/starting line; I-B, inter-block; RT, reaction time; FBT, front block time; RBT, rear block time; TBT, total block time; FPF, front peak force; RPF, rear peak force; H_FPF, horizontal front peak force; V_FPF, vertical front peak force; H_RPF, horizontal rear peak force; V_RPF, vertical rear peak force; ATF, average total force; FF_impulse_, front force impulse; RF_impulse_, rear force impulse; Total F_impulse_, total force impulse; Ratio_front, Ratio of horizontal to resultant force impulse of front leg; Ratio_rear, Ratio of horizontal to resultant force impulse of rear leg; NAHEP, normalized average horizontal external power; H_BV, horizontal block velocity; 5 m, time at 5 meters; 10 m, time at 10 meters; NorSL_1_, first stride length normalized to leg length; NorSL_2_, second stride length normalized to leg length.

The front block/starting line distance was significantly lower (-3.2 cm) in the AS vs. the US whereas the inter-block distance was significantly greater (+9.2 cm). Furthermore, at the set position, the rear hip and the rear knee joint angles were significantly greater in the AS vs. the US (+6 and +5 degrees, respectively) while the opposite was found at the front hip and knee joint angles (-4 and -3 degrees, respectively). The rear ankle joint angle was significantly lower in the AS vs. the US (-2 degrees); no significant difference was found for the front ankle joint angle. Results also showed that, during the pushing phase, several kinematic and kinetic variables were significantly greater in the AS vs. the US namely, the FBT (+0.02 s), the RPF (+1.63 N/kg), the H_RPF (+0.43 N/kg), the RF_impulse_ (+0.10 Ns/kg), the Total F_impulse_ (+0.17 Ns/kg), the Ratio_rear (+0.01), H_BV (+0.14 m/s), and the NAHEP (+0.03). No significant differences were found for the other variables during the pushing phase. In the acceleration phase, the times at 5 m and 10 m were significantly lower in the AS vs. the US (-0.05 and -0.04 s, respectively). NorSL_1_, and NorSL_2_ were significantly larger in the AS vs. the US (+0.03 and +0.04, respectively). The effect size was large (>0.14) for all the significantly different variables except for FBT, NorSL_1_, and NorSL_2_ where the effect size was medium (>0.10 effect size <0.12).

As shown in Table 3, the results of the mixed-design ANOVA (group by condition) revealed a significant main effect for condition (US and AS) by group (brachycormic, metricormic and macrocormic) interaction for a number of variables. The effect size for all the significantly different variables was large (>0.14). Post hoc analysis showed significantly greater mean inter-block distance in the AS vs. the US in the brachycormic, metricormic and macrocormic groups by respectively +7.4 cm, +9.9 cm and +10.0 cm (P<0.001 for all); in the AS condition, RPF was significantly greater in the brachycormic and the metricormic group (+1.91 N/kg, P = 0.001 and +2.32 N/kg, P<0.001, respectively), but not in the macrocormic group. H_RPF was significantly greater for brachicormic group (+0.45 N/kg, P = 0.031) and the metricormic group (+0.71 N/kg, P<0.001). In the AS V_RPF was significantly greater in the metricormic group vs. the US (+0.47 N/kg, P = 0.001) and the V_FPF was significantly higher in the macrocormic group (+0.52 N/kg, P = 0.013). RF_impulse_ was also significantly greater in the metricormic group (+0.23 Ns/kg, P<0.001) in the AS vs. the US.

The estimation of a regression model for each performance variable using the difference (Delta) in the anteroposterior block distances between the two conditions (US and AS) as predictors and the Delta in the performance variable as dependent variable, evidenced some statistically significant relationships which were summarized in Table 4.

**Table 4:** Multiple linear regression model estimated using the difference (Delta) in the anteroposterior block distances between the two conditions (US and AS) as predictors and the delta in the performance variable as the dependent variable.

	Delta FB/SL distance	Delta I-B distance	Adj. R^2^
Delta RPF	r = -0.35; P = 0.024	r = -0.26; P = 0.102	0.11
Delta H_RPF	r = -0.31; P = 0.047	r = -0.21; P = 0.175	0.07
Delta V_RPF	r = -0.37; P = 0.017	r = -0.19; P = 0.246	0.10
Delta RF_impulse_	r = -0.34; P = 0.027	r = -0.03; P = 0.836	0.08
Delta Total F_impulse_	r = -0.50; P < 0.001	r = -0.10; P = 0.545	0.25
Delta Rear hip	r = 0.33; P = 0.036	r = 0.62; P < 0.001	0.36

US, usual sprinter’s block setting; AS, anthropometry-driven block setting; FB/SL, front block/starting line; I-B, inter-block; Adj. R^2^, adjusted coefficient of determination; r, partial correlation coefficient; RPF, rear peak force; H_RPF, horizontal rear peak force; V_RPF, vertical rear peak force; RF_impulse_, rear force impulse; Total F_impulse_, total force impulse.

## Discussion

The first aim of this study was to investigate the effect of two different block setting conditions (US and AS) on kinematic and kinetic performance outcomes during the sprint start in well-trained sprinters focusing on block phases (set position and pushing-phase), and the early acceleration phase (times at 5 m and 10 m, first and second stride lengths). Results showed that an anthropometry-driven block setting condition (AS) based on the sprinter’s leg length [11] is associated with several statistically significant changes in postural parameters at the set position, as well as in kinetic and kinematic variables at the pushing and acceleration phases in comparison with the sprinter’s usual block setting, leading to improved performance.

When using the US condition, which was based on the individual sprinter’s preferences, all participants in the sample adopted an inter-block distance (27.6 ± 2.4 cm) classifiable as a bunched start, suggesting that sprinters prefer a low anteroposterior distance between the front and rear foot. This is in agreement with recent studies [15,18] showing that sprinters with different ability levels chose an anteroposterior inter-block distance from 25 cm to 30 cm on the basis of their sensations. Nevertheless, the bunched start has been demonstrated to be the least efficient from a biomechanical perspective because less force is exerted on the starting blocks with a reduction in block velocity [12,21,42].

Several studies (2,7,11,14,18,21,22,25–27) highlighted that the increase in inter-block distance is associated with improved performance in several kinetic and kinematic variables linked to the sprint start (i.e., a greater contribution of total force generation and force impulse from the rear leg and higher block clearing velocities). On the other hand, in the AS condition, which was based on the individual sprinter’s leg length, sprinters decreased the front block/starting line distance (-6.56%) and increased the inter-block distance (+25.02%). Such variations in the anteroposterior block distances in the AS condition lead to a postural adaptation at the set position resulting in a decrease in the front hip, front knee and rear ankle joint angles and an increase in the rear hip and rear knee joint angles (Table 3). Based on the literature [3,4,17,43,44], it is reasonable to assume that these postural changes at the set position in the AS condition compared to the US condition could be associated with an improvement in performance in the subsequent pushing phase such as greater RPF, H_RPF, RFI, Ratio_rear, Total F_impulse_ and NAHEP. In fact, the resulting joint angles (front/rear) in the AS condition were similar to those reported in sprinters with ability levels ranging from national-level to world-class [3,4,17]. Consistently, scientific data also showed that the front hip and knee as well as the ankle joint angles were found to be smaller in faster than slower sprinters, allowing for the stretch-reflex of the hip extensor and the soleus muscles and the greatest velocity when leaving the blocks [43,44].

A recent study identified an important role of the rear hip joint angle to assist the generation of NAHEP during the block phase [3]. This is supported by results of regression analysis in the current study showing that the difference between the US and AS conditions in the anteroposterior front/starting line and inter-block distances predicted the difference between the two conditions in the rear hip angle (*R*^2^ = 0.39 for both distances). This suggests that the action of the rear hip extensor can be enhanced by adjusting the anteroposterior block distances. This finding is supported by a study by Slawinski and colleagues [12] reporting changes in the rear and front hip angular velocity among three different inter-block distances (bunched, medium and elongated). What is more, in the AS condition, the more flexed front hip, front knee and rear ankle joint angles as well as the shorter front block/starting line distance led sprinters to assume a lower crouched set position, (i.e., the centre of mass is closer to the ground). In the literature it is reported that a crouched set position is able to generate greater H_ BV [7,18].

From a biomechanical point view, the ability to leave the blocks at a high velocity depends on the horizontal force impulse on the blocks during the pushing phase [7]. In accordance with classic mechanical physics, impulse is equal to the product of force and time. Consequently, a higher block velocity could either be due to an increase in the net propulsion force generated or to the push duration. In our sample, no significant difference in the duration of the applied force (TBT) was found between the two starting conditions, thus the increase associated with the AS in H_BV (+4%) was due to an increased force production, not to an increase in the duration of the push against the blocks. The H_BV is a commonly measured variable when evaluating sprint start performance [6,10,11,22]. However, we agree with the statement of Bezodis and colleagues [2] that the NAHEP best describes the sprint start performance because it summarizes in a single parameter how much a sprinter is able to increase their velocity and the amount of time duration to achieve this. In our study, results showed a greater NAHEP (+6%) in the AS vs. the US indicating that anthropometry-driven anteroposterior block distances may assist sprinters in translating their centre of mass in the horizontal direction.

In line with previous studies underlining the importance of the rear leg [1,3,7,16,18] in the sprint start, another important result in the current study was the significant increase observed in the AS condition for several kinetic parameters in the rear block such as the RPF, RF_impulse_ and the Ratio_rear by respectively +12.47%, +7.19%, and +1.32%. It is likely that the greater force generated by the rear leg allowed sprinters to achieve significantly greater H_BV. According to Slawinski et al. [7], the ability of faster sprinters to leave the blocks at a higher velocity depends on the rear block total force and the rate of force development.

When considering the peak force components on the front and rear blocks, we found significantly higher horizontal and vertical peak forces at the rear block in the AS vs. the US (+8.68% and +4.54%, respectively. However, the difference in the mean value on peak force between the front and the rear blocks was -3.53 N/kg (-21.26%) and -5.06 N/kg (-30.66%) in the AS and US conditions, respectively. A similar pattern was observed for the Total F_impulse_ which was -2.17 Ns/kg (-60.95%) and -2.19 Ns/kg (-62.93%) in the AS and US conditions, respectively. The finding of a smaller force difference between the rear and front legs in the AS condition suggests that sprinters are able to get a more balanced force generation between the rear and front legs. Although results showed that sprinters generated higher FPF than RPF in both conditions, in the AS condition the RPF was greater than in the US condition. Recent studies have reported that the generation of greater forces against the rear block was the strongest predictor of sprint start performance, suggesting that forces at the rear block need to be maximised to increase performance [16,19].

In our study, the results of the regression analysis showed that the difference in the front block/starting line distance between the AS and US conditions is able to predict the difference between the two conditions in RPF, the horizontal and vertical components of RPF, as well as RFI (Table 4). These findings suggest that the front block/starting line distance is an important predictor of the ability to generate greater rear block force. On the other hand, results of the present study showed that the difference between the AS and US conditions in the front block/starting line distance is not able to predict the difference between the two conditions in the Ratio_rear. This suggests that the reduction of the front block/starting line distance does not increase the ability to direct the forces in a more horizontal direction. This is in line with a recent study [16] highlighting that the ratio of horizontal to resultant impulse against the rear block was less important in predicting sprint start performance because of its low correlation with block phase performance (standardized regression coefficient = 0.010).

The changes of the lower limb joint angles at the set position showed in the AS condition and subsequent improvement in several kinetic and kinematic parameters measured in the pushing phase, might explain the significant differences between the two conditions in the acceleration phase. In fact, several studies underpinned the importance of the block phase in the subsequent sprinting times at 5 m and 10 m as well as in the first two step lengths [6,7,9,12]. In our sample, the AS condition was associated with a decrease in the times at 5 m and 10 m (-3.59% and -2.32%, respectively) and an increase of the first and second stride lengths (+2.67% and +3,36%, respectively) vs. the US condition. This is in agreement with previous studies showing that the contributions of the hip and ankle joints to force production play an important role in the acceleration phase of the sprint start [3,6,15,45–47].

The findings of our study expand on previous findings by showing that a number of postural, kinematic and kinetic parameters of the sprint start change when setting the starting blocks according to anthropometry causing sprinters to adopt a medium start. In addition, the findings highlight the importance of the front/starting line distance as predictor of certain kinetic and kinematic variables. However, further studies are needed to better understand how the kinetic and kinematic parameters are related to this block distance. Interestingly enough, modifying the anteroposterior block distances, allowed for an immediate improvement of performance in the sprinters with no period of familiarization. However, achieving an optimal automatized movement is a long-term process. Accordingly, further studies should investigate the retention of improvement of performance after at least 24/48 hours.

The second aim of the present study was to assess whether an interaction exists between the two block setting conditions (US and AS) and the body proportionality of the sprinters (brachycormic, metricormic and macrocormic) affecting the kinematic and kinetic parameters of sprint start. In other words, the present study tested if the kinematic and kinetic parameters of the sprint start were different between the US and AS conditions depending on the body proportionality of the sprinters.

It is important to underline that the three Cormic Index groups were similar in age, sprinting experience, several anthropometric parameters (e.g. BMI, lower limb circumferences), SLJ-relative (Table 1). All of the measured kinematic and kinetic parameters in the US condition were similar as well (Table 2). This suggests that the three groups were largely comparable. In the US condition the three groups adopted a similar inter-block distance whereas the front block/starting line distance was lower in the metricormic and macrocormic groups in comparison with the brachycormic group (Table 2). Actually, the front block/starting line distance increased with the mean trunk length (sitting height) of the sprinters (Tables 1 and 2). Taken together, these findings suggest that body proportionality has some effect on the front block/starting line distance when the sprinter adopts a block setting on the basis of personal sensation.

When considering the interaction effect between the three Cormic Index groups over the two block setting conditions, a significant effect was found for the inter-block distance but not for the front block/starting line distance. This may be explained by the following: first, the finding of a significantly lower limb length in the brachycormic vs. metricormic and macrocormic groups in the presence of similar stature (Table 1); second, the lower limb length was the parameter used to calculate the anteroposterior block distances in the AS condition; third, in the US condition the three Cormic Index groups chose similar inter-block distances, while the front block/starting line distance increased together with the trunk length (Tables 2 and 3). Intriguingly, it was found that sprinters modified the joint angles at the set position in the US and AS conditions irrespective of their body proportionality. In fact, the changes in the front and rear lower limb joint angles were consistent across the brachycormic, metricormic and macrocormic groups.

A statistically significant group by condition interaction was also found in a number of performance outcomes (Tables 2 and 3). In particular, the metricormic group showed an increase in the AS condition for the RPF, (+16.52%), the H_RPF and the V_RPF (+13.12% and 11.11%, respectively), the RF_impulse_, (+15.54%) (all significant at P<0.05); a significant increase was also found in the brachycormic group for the RPF (+14.99%) and the H_RPF (+9.82%); a significant increase in the V_FPF in the macrocormic group (+7.90%). These findings show that the AS condition is able to drive important improvements in kinetic variables measured in the pushing phase only for the metricormic and in the brachycormic groups. These findings are not attributable to differences in lower limbs muscle mass or strength, because thigh and calf girth as well as performance in the standing long jump expressed relative to the leg length were similar in the three Cormic Index groups (Table 1). Taken together, the above findings suggest that the anthropometry-driven condition used in this study is not best suited for macrocormic sprinters, while the sprinters with an intermediate proportionality between the trunk and the lower limbs (i.e. the metricormic sprinters) are those who are more likely to receive extensive benefits in the kinetics of the rear leg from a block setting based on their lower limb length.

A limitation of this study is the relatively small size of the three Cormic Index groups, making it difficult to generalize our findings to performance outcomes associated with trunk and lower limb proportionality. Moreover, 2D kinematic measurement was used, which is well suited to data acquisition in a number of settings [48]. However, accurate analysis of joint angles and the two first stride lengths would have benefit from 3D acquisition [49]. Accordingly, the values of the stride lengths and joint angles presented herein should be interpreted with caution as per the presence of the parallax error and the lens distortion error which have occurred in our set-up using a 2D kinematic measurement. Despite these limitations and utilizing instrumented starting blocks, the current data provides important biomechanical evidence to further understand the influence of the set position on sprint start performance and on the importance of the rear leg action.

Taken together, the findings presented in this study show that a block setting position, calculated on the basis of a proportion of the leg length allows sprinters to improve performance in both the block phase and early acceleration, thereby confirming the hypothesis that considering the athlete’s body dimensions when calculating block setting is beneficial to the sprint start. In view of these results, future research is needed to adjust the anthropometry-driven condition investigated in this study in relation to the body proportionality of the sprinter to find the optimal anteroposterior block distances.

## Conclusion

In conclusion, the current study confirmed the role played by an anthropometry-driven block setting on the starting block performance and underpins the relevance of body proportionality in calculating personalized anteroposterior block distances. The results obtained in the present study provide new relevant information that may represent the starting point for future studies aimed to develop new guidelines for helping coaches and athletes to identify the ideal personal anteroposterior block distances. From a practical standpoint, the results of this study should encourage coaches to pay more attention to the anthropometric characteristics of their athletes when searching for a more effective block start position. It would seem that a good starting point for further exploration of this field of research would be the inclusion of the Cormic Index in the identification of an individual block settings. Moreover, current findings showed that a key kinetic and kinematic determinant of the rear block performance is the front block/starting line distance. Therefore, further research is required to elucidate the effect of this distance with kinetic and kinematic parameters to more completely understand its influence on sprint start performance.

## Supporting information

S1 File Database. (XLSX)
